# Biological and targeting differences between the rare *KRAS A146T* and canonical *KRAS* mutants in gastric cancer models

**DOI:** 10.1007/s10120-024-01468-8

**Published:** 2024-01-23

**Authors:** Elisabetta Puliga, Chiara De Bellis, Sandra Vietti Michelina, Tania Capeloa, Cristina Migliore, Claudia Orrù, Gian Luca Baiocchi, Giovanni De Manzoni, Filippo Pietrantonio, Rossella Reddavid, Uberto Fumagalli Romario, Chiara Ambrogio, Simona Corso, Silvia Giordano

**Affiliations:** 1 Department of Oncology, University of Torino, Candiolo, Italy; 2 Candiolo Cancer Institute, FPO-IRCCS, Candiolo, Italy; 3 Department of Molecular Biotechnology and Health Sciences, Molecular Biotechnology Center, University of Torino, Via Nizza 52, 10126 Turin, Italy; 4 Department of Clinical and Experimental Sciences, University of Brescia, Brescia, Italy; 5 Department of Surgery “Santo Spirito Hospital”, ASL-AL, Rome, Italy; 6 Section of Surgery, Department of Surgical Sciences, Dentistry, Gynecology and Pediatrics, University of Verona, Verona, Italy; 7 Medical Oncology Department, Fondazione IRCCS Istituto Nazionale Dei Tumori, Milan, Italy; 8 Department of Oncology, University of Torino, Orbassano, Italy; 9 Digestive Surgery, European Institute of Oncology, IRCCS, Milan, Italy

**Keywords:** Gastric cancer, *KRAS* mutants, RAS downstream signalling, Oncogene addiction

## Abstract

**Background:**

Gastric cancer (GC) is the third leading cause of cancer-related death worldwide, with a poor prognosis for patients with advanced disease. Since the oncogenic role of *KRAS* mutants has been poorly investigated in GC, this study aims to biochemically and biologically characterize different *KRAS-mutated* models and unravel differences among *KRAS* mutants in response to therapy.

**Methods:**

Taking advantage of a proprietary, molecularly annotated platform of more than 200 GC PDXs (patient-derived xenografts), we identified KRAS-mutated PDXs, from which primary cell lines were established. The different mutants were challenged with KRAS downstream inhibitors in in vitro and in vivo experiments.

**Results:**

Cells expressing the rare *KRAS A146T* mutant showed lower RAS-GTP levels compared to those bearing the canonical *G12/13D* mutations. Nevertheless, all the KRAS-mutated cells displayed *KRAS* addiction. Surprisingly, even if the GEF SOS1 is considered critical for the activation of *KRAS A146T* mutants, its abrogation did not significantly affect cell viability. From the pharmacologic point of view, Trametinib monotherapy was more effective in *A146T* than in *G12D*-mutated models, suggesting a vulnerability to MEK inhibition. However, in the presence of mutations in the PI3K pathway, more frequently co-occurrent in A146T models, the association of Trametinib and the AKT inhibitor MK-2206 was required to optimize the response.

**Conclusion:**

A deeper genomic and biological characterization of *KRAS* mutants might sustain the development of more efficient and long-lasting therapeutic options for patients harbouring *KRAS*-driven GC.

**Supplementary Information:**

The online version contains supplementary material available at 10.1007/s10120-024-01468-8.

## Introduction

Recent integrated genomic profile analyses of the major cancer mutation databases have shown that approximately one out of seven human cancers harbours *KRAS* gene alterations, making it one of the most frequently mutated genes [1, 2].

The KRAS protein is a small GTPase that cycles between an inactive GDP-bound “OFF state” and an active GTP bound “ON state”. The turn OFF/ON transition of the GTPase usually happens in response to mitogenic signals and is assisted by guanine-nucleotide exchange factors (GEFs) such as SOS1/SOS2 [3]. GTP bound KRAS drives MAPK pathway activation, ultimately leading to cell proliferation. A single amino acid change at specific codons can convert this protein into an oncogenic driver, usually impairing its GTPase activity [3], thus abrogating the return to an OFF state. Although *KRAS* mutants display different features in terms of frequency among cancers, impact on KRAS biochemical activity and oncogenic potential, they have been usually classified as a homogenous group [4]. On the contrary, it has been shown that distinct mutant forms of KRAS (*KRAS A146T* vs *KRAS G12D*) have different oncogenic potency and are able to impact on diverse tissue-specific signalling pathways [5]. In this context, based on structure–function studies on how *KRAS* mutants promote KRAS activation and influence its interaction with different effectors, mutants can be classified into four classes [6]. Class I, comprising *KRAS Gly12* mutations, is endowed with impairment of the intrinsic GTPase activity, avoiding GTPase-activating proteins (GAPs) capability to accelerate GTP hydrolysis. In Class II (*Gly13*, *Lys117* and *Ala146*) the steady-state levels of active KRAS are increased by faster nucleotide exchange. Class III includes mutations, such as *Q61H*, that have a hybrid mechanism, interfering with both GAP and intrinsic GTP hydrolysis and modestly increase the nucleotide exchange. Finally, Class IV mutations haven’t been fully characterized yet.

The frequency of *KRAS* mutations is variable across different cancer types, with mutation rates up to 88% in pancreatic ductal adenocarcinoma (PDAC), 45–50% in colorectal cancer (CRC) and 30–35% in lung adenocarcinoma [1, 2, 7]. Lower mutation frequency has been observed in gastric (9%) cervical (6.6%), prostate (5%) and oesophageal cancer (2%) [8–12].

The prognostic significance of distinct *KRAS* activating mutations has been evaluated in different cancer contexts. For instance, PDAC patients harbouring the *G12D* mutation display a worse overall survival (OS) compared with those bearing the *G12R* mutation [13]. Consistently, multiple independent analyses of CRC large cohorts have correlated *G12V* mutation with a major risk of recurrence or death [14]. In addition, KRAS-mutated alleles differently affect therapeutic responses [15]. In the context of target therapies, *G12D* and *G12S KRAS* non-small cell lung cancer (NSCLC) patients treated with EGFR TKIs showed promising response rates compared with *G12C* and *G12V* KRAS-bearing patients [16].

As oncogenic mutations can be present in one or in both alleles, a few reports have interrogated the effect of the presence of the *KRAS* WT allele together with the mutated counterpart. In this context, it has been shown that mutant to wild-type alleles ratio critically impacts tumour fitness, since the allelic imbalance might influence the response to anticancer therapies aimed at inhibiting RAS/MEK signalling [17].

A deeper knowledge of mutant KRAS proteins and their “oncogenic modus operandi” breaks in the concept of the importance to develop *KRAS* mutants–selective therapeutic strategies. Compelling evidence supporting the efforts to design allele-specific therapies and to target the peculiar biochemical/biological features of each mutant, comes from the recent generation of two specific KRAS G12C inhibitors (Sotorasib and Adagrasib) covalently binding cysteine 12 within the switch-II pocket of KRAS G12C and locking KRAS in the inactive state, thus arresting cell proliferation [18, 19]. In 2021, based on a phase 2 trial, the U.S. Food and Drug Administration (FDA) accelerated the approval of Sotorasib as the first KRAS G12C blocking drug for treatment of adult patients with NSCLC [20]. Afterwards, FDA granted breakthrough therapy designation to Adagrasib as a potential treatment option for NSCLC patients with KRAS G12C mutations after previous systemic therapy [21]. Recently, a new potent and selective non-covalent KRAS G12D inhibitor (MRTX1133) has been identified, reaffirming the importance and feasibility of selectively targeting *KRAS* mutants [22].

Gastric cancer (GC), ranking among the top five malignant tumours worldwide in terms of incidence and mortality [23], is a highly heterogeneous disease, with geographical variants and different molecular landscapes [8, 24]. Although improvement of treatment options allowed achieving a survival benefit, the prognosis remains poor. Since the *KRAS* gene is frequently amplified in GC (6%, cBioportal), different reports have studied the response to MEK inhibition in amplified models [25]. Due to the lack of studies investigating the role of the diverse *KRAS* mutations in GC, taking advantage of a proprietary, molecularly annotated platform of GC PDXs (patient-derived xenografts)[26] we aimed to: i) molecularly and biochemically characterize different *KRAS-mutated* models and ii) unravel *KRAS* mutants’ differences in terms of response to therapy, with a particular focus on the rare and poorly studied *A146T* mutation.

## Material and methods

### Animals and preclinical trials

NOD (non-obese diabetic)/SCID (severe combined immunodeficient) mice were purchased by Charles River (Milan, Italy). Guidelines for Care and Use of Laboratory Animals (‘Animal Research: Reporting of In Vivo Experiments’ (ARRIVE) standards) were followed during the investigation. All animal procedures were approved by Ethical Commission of the IRCC in Candiolo and the Italian Ministry of Health. Gastric PDX generation was performed as described in [26]. PDXs were passaged and expanded for > two generations until production of a cohort of mice. Established and randomized tumours (average volume 250/300 mm^3^; *N* = 5) were treated for the indicated days with the following regimens (either single agent or combination): vehicle (saline) per os; Trametinib 1 mg/kg, daily, per os; MK-2206 100 mg/kg, three times per week, per os. Tumour size was evaluated once a week by calliper measurements and approximate volume of the mass was calculated using the formula 4/3*π*(*D*/2) (*d*/2)^2^, where D is the major tumour axis and d is the minor tumour axis. Statistical significance: ns = not significant; **p < 0.01; ***p < 0.001. PDX models data and metadata will be openly available in PDX Finder (https://doi.org/10.1093/nar/gky984, pdxfinder.org) and in the EurOPDX data portal (http://dataportal.europdx.eu).

### Primary cells culture and in vitro experiments

GC primary cells (GTR0245, GTR0249, GTR0164, GTR0213, GTR0207, GTR0128) were derived from PDXs as described in [26]. LS1034 and SNU81 cell lines were obtained from ATCC (Manassas, VA, USA) and the Korean Cell line bank (Seoul, Korea), respectively. The genetic identity of the in vitro-derived material with the original tumour has been verified by short tandem repeat profiling (Cell ID, Promega Madison, WI, USA). Exome analysis and Sanger method have been applied for the detection of *KRAS*, *PI3KCA* and *PTEN* mutations. For growth curve assay, cells were seeded in quadruplicates in 96-well culture plates (6000/8000 cells/well depending on the cell lines) either in the presence or in the absence of Trametinib, MK-2206 (Selleckchem Chemicals) or the combination for 72 h. At the end of the experiment, cells were stained with the crystal violet dye and colorimetric measurement was performed at 570 nm with a Multilabel Reader (PerkinElmer). GTR0207 model was not included in viability and silencing experiments because of the difficulty to propagate this primary cell line in culture for more than few days.

### Gene silencing

Lentiviruses were produced as described in [27]. GTR0245, GTR0249, GTR0164, GTR0213 and GTR0128 primary cells were transduced either with a mixture of lentiviruses containing two KRAS short hairpin RNAs (shRNA; Sigma, #33260, #352609) or PLKO empty vector. Twenty-four hours after transduction, cells were seeded and tested for cell viability 72 h later. At the end of the experiment, cells were stained with the crystal violet dye and colorimetric measurement was performed at 570 nm with a Multilabel Reader (PerkinElmer). For *SOS1* silencing, GTR0245, GTR0249, GTR0213 and GTR0128 cells were transfected using Lipofectamine 2000 (Thermo Fisher Scientific) with 20 nmol/L of synthetic SOS1 (ON-TARGET plus Human SOS1 (6654) siRNA, Dharmacon reagents) or Control (AllStars Neg. Control siRNA, Qiagen) siRNAs according to standard methods. Seventy-two hours later, cell viability was evaluated by CellTiter Glo (Promega Inc, Madison, WI, USA).

### Gene expression analysis by qRT-PCR

For the evaluation of *KRAS* and *SOS1* gene expression, total RNA was extracted using Maxwell® RSC miRNA from Tissue and Plasma or Serum AS1460 (Promega) and reverse transcribed into complementary DNA using the High-Capacity complementary DNA Reverse Transcription Kit (Applied Biosystems, Carlsbad, CA, USA) and random primers, according to the manufacturer’s protocol. Complementary DNA (500 ng) was amplified by Real-time qPCR using the Ssoadvanced universal probes super mix (Bio-Rad), according to the manufacturer’s protocol. Real time qPCR was performed using the following primers: ACTB (assay ID: Hs99999903_m1); KRAS (assay ID Hs00364283_g1) and SOS1 (assay ID Hs00893128_m1) (Thermo Fisher Scientific).

### RAS G-LISA assay

The RAS G-LISA assay was performed using the G-LISA Ras activation (absorbance based) kit (Cytoskeleton, Inc, DENVER) according to manufacturer’s instructions. All KRAS-mutated models were starved with 0.5% FBS for 24 h. 293T cells were used to normalize the levels of RAS-GTP among the KRAS-mutated models. Three independent experiments were performed for each KRAS-mutated model, except for the GTR0207 model (1 experiment performed) for the same reason mentioned above.

### Western blot analysis

Cells were treated for 6 h with the indicated drugs and concentrations (Trametinib 5 nM, MK-2206 5uM or the combo treatment). Whole-protein extracts were prepared using Laemmli buffer and quantified using the BCA Protein Assay kit (Pierce, Rockford, IL, USA). Primary antibodies: phosphorylated-p44/42MAPK (Thr202/Tyr204), phosphorylated AKT (Ser473) (Clone D9E), total AKT, and ERK were purchased from Cell Signaling while the Vinculin (1931) from Sigma. Secondary antibodies were from Amersham and the detection was performed with ECL system (Amersham, UK).

### Immunohistochemestry and fluorescence in situ hybridization

Tumour xenografts from mice receiving 2 days treatments with Trametinib, MK-2206 and combo were harvested for IHC analysis. Sections were cut (5 µm) and PS6 immunohistochemical analysis was carried out. Briefly, sections were deparaffinized and hydrated. Antigen retrieval was performed using Cytrate buffer solution, at 95 °C for 40 min. Endogenous peroxidases were quenched using metanol–0.3% H_2_O_2_. Primary antibody used PS6 (Ser235/236) (D57.2.2E) from Cell Signalling (Danvers, MA, USA). Diluted antibody was applied to the sections overnight and then detected using anti-rabbit reagent and DAB Substrate (Thermo Fisher). Tissues were counterstained with Harris’ hematoxylin, dehydrated, cleared, and coverslipped. To determine *KRAS* gene zygosity, dual-color FISH was performed on GTR0245 PDX 4 µm thick section using the LSI KRAS SpectrumGold and CEN12 probes (Abbott Molecular).

### Statistical analysis

Statistical significance was performed using the two tailed Student’s t test and the two-way ANOVA Bonferroni’s multiple comparison to compare differences between experimental conditions (GraphPad Prism 9 software).

## Results

### The GC PDX platform recapitulates the distribution of KRAS mutants in the TCGA cohort

**Fig. 1:**
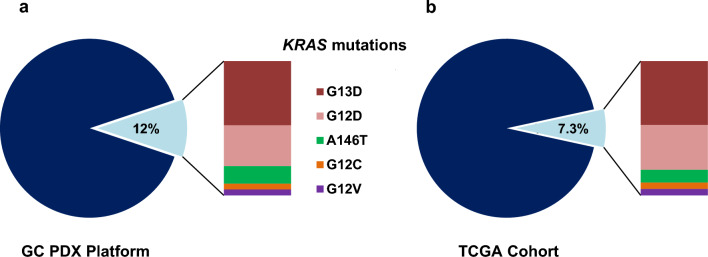
The GC platform is representative of the *KRAS* mutants in the TCGA cohort. Pie charts showing the percentage of KRAS-mutated patient-derived xenografts (PDX) present in the PDX Gastric Cancer platform (**a**) and in the TCGA cohort (**b**)

To capture the biological and biochemical characteristics of the different *KRAS* mutants in our GC PDX platform, we first evaluated their distribution among the 200 profiled available models. *KRAS* mutations have been detected in 23 PDXs (12% of PDXs); among them, the most frequent mutations were *G13D* (11, 47.8%), *G12D* (7, 30.4%), *A146T* (3, 13%), *G12C* (1, 4.3%) and *G12V* (1, 4.3%) (Fig. 1a). The distribution of *KRAS* variants was found in line with the percentage of mutants reported in the TCGA cohort (7.3% *KRAS-mutated* patients of which 47.6% *G13D*, 33.3% *G12D*, 9.5% *A146T*, 4.7% *G12C*, 4.7% *G12V* mutants), underlining the potential of this PDXs GC collection to capture *KRAS* mutational status in this malignancy (Fig. 1b). Interestingly, as previously reported [26], the frequency of *KRAS* mutations is increased in PDXs due to higher engraftment rate but the ratio of the different mutants is not altered.

### GC KRAS-mutated cells display different KRAS-GTP levels but similar addiction to the KRAS gene.

**Fig. 2:**
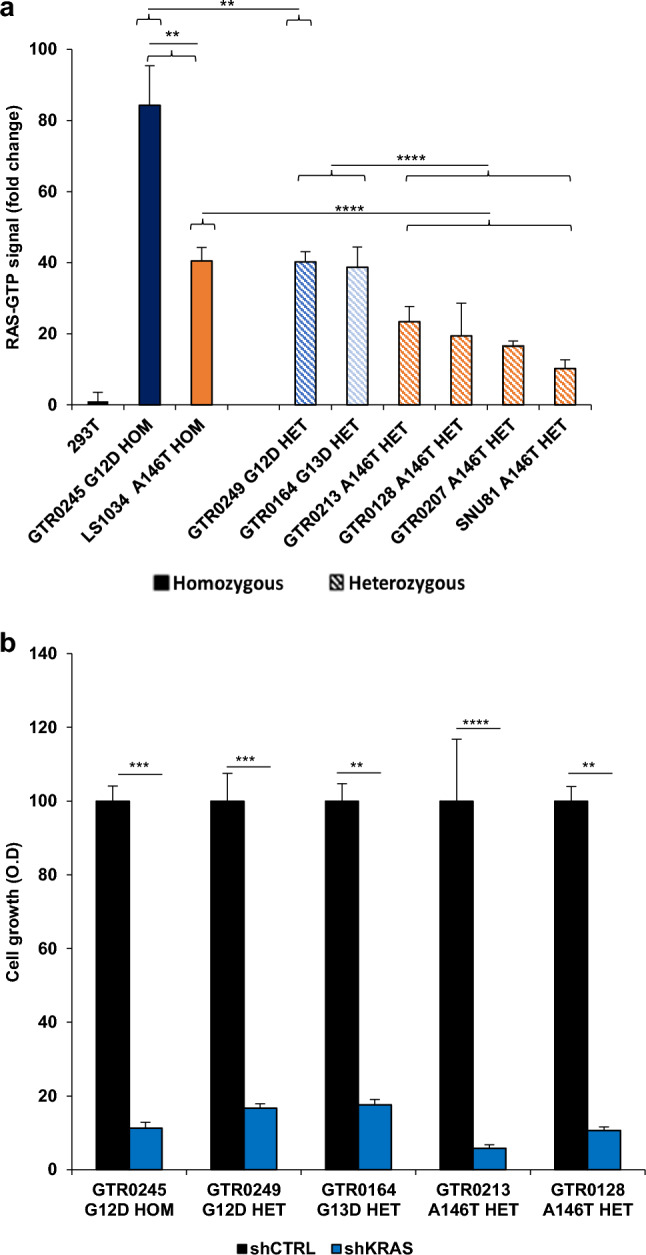
*KRAS* mutants display different levels of activation but similar addiction to the *KRAS* oncogene. **a** Bar graph comparing RAS-GTP levels in KRAS-mutated models. Values in fold change of RAS-GTP levels are relative to 293T cells (WT for *KRAS*). Filled bars indicate homozygous mutants; dashed bars represent heterozygous mutants. **b** Cell viability upon *KRAS* silencing was evaluated in five KRAS-mutated gastric cancer models using crystal violet staining. Both histograms show mean ∓ SD of at least three independent experiments. *sh* short hairpin; (Student’s *t* test) *p < 0.05; **p < 0.01; ***p < 0.001; ****p < 0.0001

It is known that gain-of-function missense mutations increase KRAS GTP levels [28]. Since the level of activation of the KRAS A146T is largely unknown, we performed G-LISA RAS activation assay (Fig. 2a) on PDX-derived primary cells (3 KRAS A146T, 2 KRAS G12D and 1 KRAS G13D models) (Suppl. Figure 1a). Since the availability of *A146T* gastric primary cellular models was limited, we included two colorectal cancer cell lines bearing this less frequent mutation (SNU81 mutated in a single allele and LS1034 lacking the WT allele). As shown in Fig. 2a, we observed that in GTR0245 cells, presenting a homozygous *G12D* mutation (Suppl. Figure 1b), the level of active KRAS was higher than in GTR0249 cells, carrying the same mutation in heterozygosis. Similarly, the presence of the *A146T* mutation in homozygosis (in LS1034 cells) resulted in a level of RAS activation higher than in the heterozygous counterpart. This result suggests that the co-existence of two mutated alleles leads to a more potent KRAS activity. Interestingly, the KRAS activation state in *A146T* mutated models, both in heterozygosis and homozygosis, was lower than that of cells mutated for *G12/13D*.

In spite of the different KRAS activation status, however, cells were equally addicted to the different mutants, as shown by in vitro silencing experiments, in which we transduced five mutated models with two different KRAS short hairpin RNAs (shRNAs) (Fig. 2b and Suppl. Fig b). In sum, our results show that, regardless the type of mutation and zygosity, KRAS-mutated cells significantly rely on the activity of this oncogene for survival, suggesting that its inhibition might lead to a therapeutic response in all the mutated cases.

### KRAS-mutated models response to KRAS downstream inhibitors depends on their intrinsic molecular landscape

Over the years, many research groups have put their efforts in tackling this ‘difficult-to-target’ oncoprotein, implementing direct and indirect strategies to target KRAS mutants. To study whether *KRAS G12D* and *A146T* mutants were able to differentially activate KRAS downstream pathways, we explored their sensitivity to the MEK-inhibitor (MEKi) Trametinib, alone or in combination with the AKT inhibitor MK-2206.

**Fig. 3:**
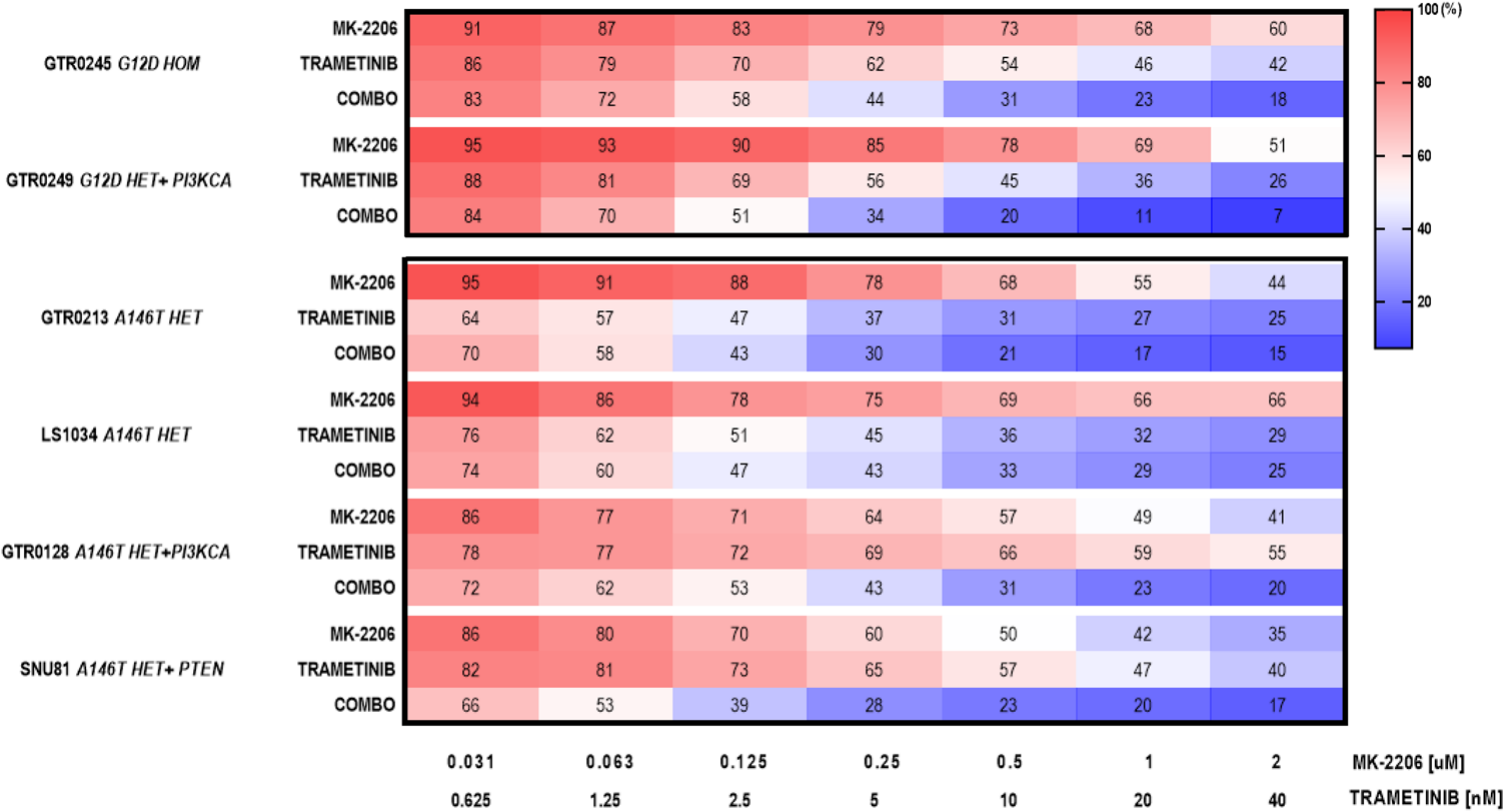
*KRAS A146T* models show a strong sensitivity to Trametinib in vitro treatment, in the absence of additional driver mutations. Heatmap showing the viability of different *KRAS* mutant PDXs treated for 72 hours with the indicated doses of the single agents Trametinib or MK-2206 or the combo. The average of three independent experiments is shown. The scale represents the percentage of viable cells

As shown in Fig. 3, while models harbouring the *G12D* mutation (GTR0245 and GTR0249) benefitted of the drug combination, a strong effect of Trametinib alone was observed in two of the *A146T* mutated models (GTR0213-heterozygous- and LS1034-homozygous-), suggesting a vulnerability for this mutant at low doses of the MEKi. On the other hand, in the GTR0128 and SNU81 models (*A146T* heterozygous mutants) the Trametinib/MK-2206 combination resulted in a remarkably increased response, likely due to the additional presence of point mutations in *PIK3CA* (GTR0128) and *PTEN* (SNU81) genes, respectively (Table 1). Interestingly, the interrogation of gastric cancer databases showed that the co-occurrence of *KRAS* and *PIK3CA* pathway mutations is around twofold more frequent for *A146T* than for the other mutants (66% vs 23%, Suppl. Figure 4).

**Table 1:** Summary of relevant mutations detected in *KRAS A146T* models

Sample name	Source	Gene	Mutation	AA change	Classification	All fraction/zigosity
GTR0128	Exome	*KRAS*	p.A146T	c.436G>A	Missense	0.44
		*PIK3CA*	p.E542K	c.1624>A	Missense	0.53
SNU81	Cosmic	*KRAS*	p.A146T	c.436G>A	Missense	Heterozygous
		*PTEN*	p.R130G	c.389>A	Missense	Heterozygous
			p.E299ter	c.895G>T	Nonsense	Heterozygous

**Fig. 4:**
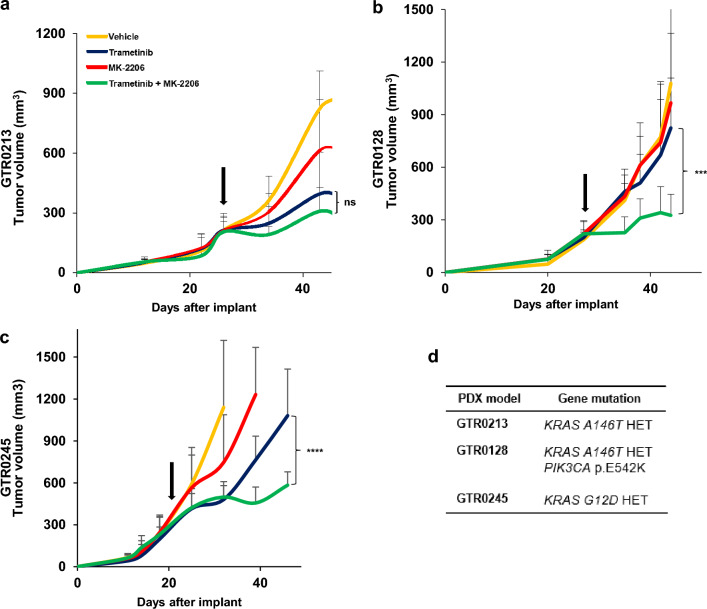
In vivo effectiveness of the single agent Trametinib in *KRAS A146T* mutants without co-occurent *PIK3CA/PTEN* mutations. **a**–**c** Tumor growth curves of mice cohorts derived from GTR0213, GTR0128 and GTR0245 PDXs, treated with the MEKi inhibitor Trametinib and the AKT inhibitor MK-2206, alone or in combination, as indicated. The different inhibitors were used at the following doses: Trametinib 1 mg/kg, daily, per os; MK-2206 100 mg/kg, 3 times per week, per os. Lines represent average tumor volumes + SD for at least 5 animals. Statistical significance was calculated using the Two-way ANOVA with Bonferroni correction. *ns* not significant, **p < 0.01; ***p < 0.001; the black arrows indicate the start of the treatment. **d** *KRAS* and co-occurent mutations for each preclinical model

To confirm our in vitro results and validate the sensitivity of *A146T*-mutants to Trametinib in the absence of concomitant driver mutations, we performed preclinical trials on the PDXs from which the primary cell lines have been generated (Fig. 4). Briefly, KRAS-mutated PDXs were passaged until production of a cohort of 40 mice. Established tumours (average volume, 300 mm^3^) were randomized and treated with Vehicle, Trametinib, MK-2206, either as single agents or in combination. Results confirmed the sensitivity of GTR0213 tumours (*A146T*-mutated model) to Trametinib alone, showing no statistical difference between the single arm of MEKi and the combination treatment (Fig. 4a). In agreement with in vitro experiments, the combo was the only effective treatment in the context of the GTR0128 PDX, in which the *A146T* mutation is concomitant with a *PIK3CA* mutation (Fig. 4b). Likewise, the GTR0245 model (*G12D* homozygous) showed a statistically significant difference between the Trametinib monotherapy and the combination (Fig. 4c).

**Fig. 5:**
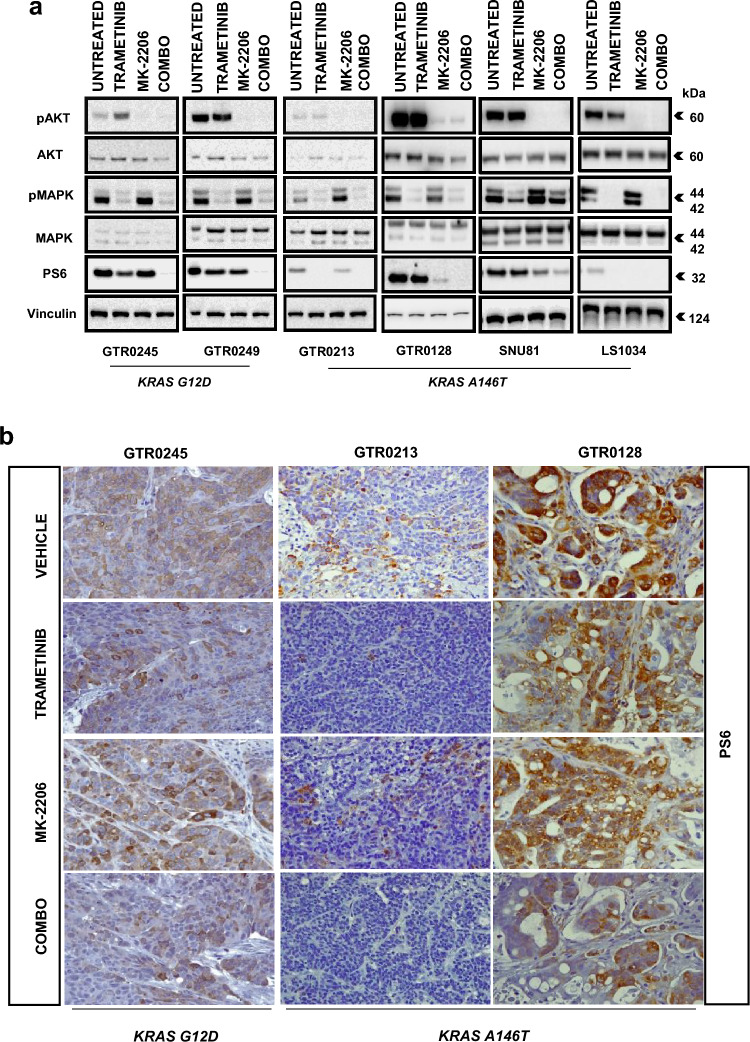
In vitro and in vivo signal transduction properties of *KRAS* mutants upon treatment with Trametinib, MK-2206 or the combo. **a** Western blot analysis of *KRAS-mutated* models upon 6 h-treatments with Trametinib, MK-2206 or the combo. Vinculin probing was used as loading control **b** PS6 immunohistochemistry staining of tumor slices obtained from mice receiving vehicle or acute treatments (2 days) with Trametinib, MK-2206 or combo. Magnification: ×40

Biochemical (Fig. 5a) and immunohistochemical (Fig. 5b) analyses confirmed the high sensitivity of two *A146T* mutated models (GTR0213-heterozygous- and LS1034-homozygous-) to Trametinib monotherapy, showing its ability to abrogate the activation of the PI3K/MAPK downstream effector S6 kinase. On the contrary, *G12D-mutated* models needed the combo treatment to show the same effect on PS6.

This striking effect of Trametinib on PS6 in GTR0213 and LS1034 *A146T*-mutated models, may be due to the previously reported [5, 29] “weakness “of this allele to induce KRAS downstream signals. Conversely, *A146T*-mutated models (such as GTR0128 and SNU81) displaying additional and “strong” driver mutations (*PI3KCA* and *PTEN* respectively) needed the combo treatment to downregulate PS6 (Fig. 5a, b).

### Inhibition of the guanine exchange factor SOS1 does not significantly affect the viability of *KRAS A146T* mutants

**Fig. 6:**
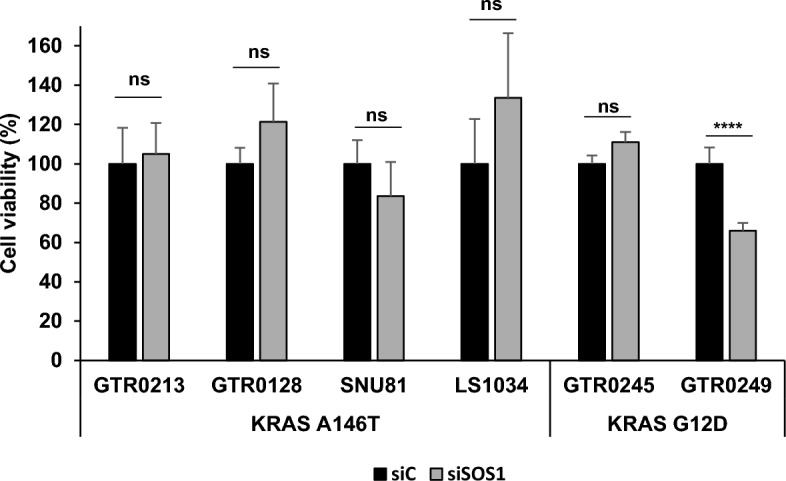
SOS1 silencing does not significantly affect viability of KRAS A146T mutants. Bar graph representing the percentage of cell viability of *KRAS-mutated* models 48 h upon transfection with SOS1 siRNA. Cell viability was measured using Cell Titer Glo cell viability assay. Bar graphs display mean ± SD; comparisons were made using Student’s *t* test; *ns* not significant; ****p < 0.0001

As already described by Poulin et al. [5], *KRAS A146T* mutants are characterized by a protein structure that does not impair the activation of the GTPase, thus promoting a high rate of intrinsic and GEF-induced nucleotide exchange. We thus investigated if the abrogation of the GEF SOS1 could differentially affect the viability of *KRAS A146T* mutants compared to the canonical *G12D* mutants. Unexpectedly, silencing experiments (Suppl. Figure 3) showed that the viability of the *A146T* mutated models was not significantly affected by *SOS1* silencing, regardless of their zygosity. The same result was obtained for the GTR0245 model, homozygous for the *G12D* mutation, while the GTR0249 (carrying the same mutation in heterozygosis) showed a modest reduction of cell proliferation upon *SOS1* silencing (Fig. 6), in agreement with what shown by Wong et al*.* [25]. Thus, our results demonstrate that, even if the GEF SOS1 is considered a key player in the activation of *KRAS A146T* mutants, its abrogation does not significantly affect their viability, suggesting that pharmacological targeting of this protein is unlikely to be effective.

## Discussion

During the past four decades, *KRAS* oncogene targeting has attracted substantial attention and efforts by researchers from all over the world. Considered as the holy grail of cancer drug discovery, because of its own characteristics, KRAS has been regarded as undruggable for years. Thanks to an unceasing and active exploration, novel insights on *KRAS* mutants, a deeper knowledge of their structure, and a consequent development of drugs for their direct targeting have been reached. The recent debut of specific *KRAS-G12C* and *KRAS-G12D* inhibitors has made breakthroughs in the development of new therapeutics, with the ultimate goal to target all KRAS mutants. Such a desirable intent has to be supported by a detailed biological and signalling profile of the different mutants.

GC is characterized by both *KRAS* gene amplification and mutation but only few studies have been focused on the role of the different *KRAS* mutants in this tumour context. We thus took advantage of our molecularly annotated GC PDX platform to derive, characterize and possibly unravel weaknesses of the less frequent *KRAS A146T* models. From PDXs, we have been able to derive primary cell lines that maintain the *KRAS* alterations observed in the primary tumour. The analysis of these GC primary cells showed that the different *KRAS* mutants display diverse RAS-GTP levels, with the A146T models presenting lower RAS-GTP content compared with *G12D/G13D* mutants. These results are in line with the experiments performed by Janakiraman et al., in which the level of RAS-GTP observed in HEK-293FT transfected with the *A146T* mutant was lower than that observed upon *G12D KRAS* transfection [29]. Nevertheless, KRAS silencing experiments indicated that all the models, independently of their mutation, relay on KRAS expression for growth and survival, giving unquestionable evidence of the addiction to the *KRAS A146T* mutant, in spite of the lower level of activation.

The recruitment of the guanine exchange factor SOS1 is a crucial step for RAS activation, given its role in the exchange of GDP for GTP [30]. Previous studies have demonstrated that SOS1 abrogation decreased the survival of pancreatic tumour cells harbouring canonical *KRAS* mutants [31]. However, according to our experiments, this observation does not turn to be true for the *KRAS A146T* mutant, since *SOS1* silencing did not decrease cell viability in these mutated models -regardless their zygosity. A possible explanation for our observation is that, as previously mentioned [6], mutants characterized by GDP/GTP hyper exchange, such as the A146T, may reach a threshold of nucleotide exchange where GEF activity is superfluous, rendering cancer cells unresponsive to GEF abrogation. Regarding *KRAS G12D* mutants we observed a significant decrease in viability in the model bearing the mutation in heterozygosis but not in the model bearing the mutation in homozygosis. This is in line with recent studies showing a stronger potential of the SOS1 inhibitor BI-3406 to limit cancer cell proliferation in NCI-H23 isogenic cells bearing a *KRAS* mutation in heterozygosis compared with the homozygous counterpart [32], making relevant the evaluation of the wild-type allele contribution upon SOS1 inhibition. Notably, the inhibitory effect of BI-3406 was not observed in *KRAS* wild-type cells not addicted to this gene [32]. Interestingly, in the case of the *A146T* mutants we did not observe any effect of SOS1 silencing neither in the presence, nor in the absence of the WT allele.

Inhibiting KRAS downstream pathways has been the most explored strategy to target KRAS oncogenic activation. In this study, treatment with Trametinib (MEK-inhibitor) and MK-2206 (AKT inhibitor) showed not superimposable responses (both in in vitro and in vivo experiments) on *KRAS G12D* and *KRAS A146T* models, highlighting the different ability of the mutants to activate KRAS downstream pathways. In the absence of “additional” driver mutations (such as those in the PI3K pathway), the *KRAS A146T* models displayed vulnerability to MEK inhibition compared with *KRAS G12D* models, further demonstrating the “weakness” of the *A146T* allele. Indeed, as suggested, due to its reduced affinity for GTP, *KRAS A146T* reasonably drives an unstable and weak downstream signal compared with *KRAS G12D*, able to induce a strong and continuous signal [29]. This hypothesis was confirmed by our experiments since Trametinib monotherapy efficiently decreased PS6 levels in *KRAS A146T* models, while the combo treatment (Trametinib + MK-2206) was required to elicit the same effect in *KRAS G12D* models. Moreover, biochemical analyses showed the failure of *KRAS A146T* models to induce pAKT rebound after AKT inhibition, a feedback effect visible in *KRAS G12D* mutants. Interestingly, we observed that the co-occurrence of the *KRAS A146T* mutation with alterations of genes of the PI3K pathway was twice as much frequent compared to that of canonical oncogenic alleles. This observation, on one side, strengthens the concept of the “weak allele” and on the other side reinforces the rationale for a combination approach simultaneously hitting MAPK and PI3K pathways.

Overall, our data underlie the importance of deepening the knowledge of the allele-specific signalling properties and their readout in terms of response to treatment. Although the use of inhibitors of RAS downstream signalling (particularly the MAPK pathway) has raised concerns due to their toxic profiles, recent studies have shown the efficacy and the tolerability of combining ERK and SHP2 inhibitors in the treatment of in vitro and in vivo models of murine and human RAS-driven pancreatic ductal adenocarcinoma [33]. Moreover, in a panel of Non-Small Cell Lung Cancer models, the association of the KRAS G12C inhibitor ARS1620 and a PI3K inhibitor was effective also in models resistant to single agent ARS1620 [34], further reinforcing the concept that using combinations of drugs targeting RAS downstream effectors is not an obsolete therapeutic approach but a challenging path still worthwhile to tread. In line, our work underlines the importance of persisting in unceasing combination approaches relying on a deep knowledge of the signalling pathways preferentially activated by the different mutants. Even if specific KRAS mutant inhibitors have generated a lot of expectations [35], their clinical advantage has been limited and interrupted by the insurgence of resistance. In this scenario, based on the ability of KRAS mutants to differentially activate the downstream pathways, it is reasonable that the use of combinations (for example with SOS1 or MEK inhibitors), tailored on the presence of a specific mutant, will be necessary to sustain the efficacy of KRAS-mutant specific inhibitors.

Specific KRAS variants show different transforming mechanisms impacting the impairment of GTP hydrolysis. Specifically, the KRAS A146 variant leads to guanine-nucleotide exchange factors (GEF)-mediated activation without impacting GAPs. Therefore, KRAS A146-driven cancers may be sensitive to Son of Sevenless protein 1 (SOS1) inhibitors, combined with a MEK-inhibitor, or with SHP2, thus reinforcing the role of MEK inhibition in these tumours even in the current drug development scenario.

Recent findings of several groups, including ours, support the existence of a functional, clinically impactful heterogeneity of *KRAS-mutated* tumours. Our work points out the need for a deeper genomic and biological characterization of *KRAS* status in tumours, such as gastric cancer, where this is not routinely evaluated. However, neither cells bearing *KRAS* amplification (6% of gastric tumours) nor *KRAS* mutations (7%) can be killed with a single *KRAS*-targeted drug. Since it is now clear that the activation mechanism of the different *KRAS* mutants is not always superimposable and that the interaction with upstream or downstream molecules is mutant-specific, it becomes critical to study in depth the biological and biochemical characteristics of each *KRAS* mutant. In this perspective, we have shown that the *KRAS* A146T mutant, being less active than the canonical ones, is more sensitive to MAPK inhibition, while it is not affected by the inactivation of SOS1 alone. On the contrary, since it is frequently concomitant with PI3K activation, the association with PI3K-specific drugs can improve the efficacy of the treatment. For all the above-mentioned reasons, we believe that our findings may be helpful in guiding drug development strategies in patients with A146T-driven gastric cancer or other solid tumours.

### Supplementary Information

Below is the link to the electronic supplementary material.

Supplementary file1 (PDF 217 KB)

## Declarations

### Animal studies

All institutional and national guidelines for the care and use of laboratory animals were followed.
