# Supplementary material for: Biological and targeting differences between the rare KRAS A146T and canonical KRAS mutants in gastric cancer models
Source: Gastric Cancer. 2024 Jan 23;27(3):473–83. doi: 10.1007/s10120-024-01468-8 (PMC11016506; doi:10.1007/s10120-024-01468-8)
Supplement: Supplementary file 1 — Supplementary file1 (PDF 217 KB) [file 10120_2024_1468_MOESM1_ESM.pdf]

ORIGINAL ARTICLE

Biological and targeting differences between the rare KRAS A146T and canonical KRAS mutants in gastric cancer models.

Elisabetta· Puliga[1,2*] Chiara De Bellis[1,2], Sandra Vietti Michelina[3] , Tania Capeloa[1,2], Cristina Migliore[1,2], Claudia Orrù[1,2], Gian Luca Baiocchi[4,5] , Giovanni De Manzoni[6] , Filippo Pietrantonio[7] , Rossella Reddavid[8] , Uberto Fumagalli Romario[9] , Chiara Ambrogio[3] , Simona Corso[1,2] and Silvia Giordano[1,2] .

[1]Department of Oncology, University of Torino, Candiolo, Italy. [2]Candiolo Cancer Institute, FPO-IRCCS, Candiolo, Italy. [3]Department of Molecular Biotechnology and Health Sciences, Molecular Biotechnology Center, University of Torino, Via Nizza 52,10126 Torino, Italy [4]Department of Clinical and Experimental Sciences, University of Brescia, Italy; [5]Department of Surgery ''Santo Spirito Hospital'', ASL-AL; [6]Department of Surgical Sciences, Dentistry, Gynecology and Pediatrics, Section of Surgery, University of Verona, Italy; [7]Medical Oncology Department, Fondazione IRCCS Istituto Nazionale dei Tumori, Milan, Italy; [8]Department of Oncology, University of Torino, Orbassano, Italy; [9]Digestive Surgery - European Institute of Oncology - IRCCS – Milan;

*Corresponding author: elisabetta.puliga@ircc.it, +39 011 993321

Supplementary Figure 1

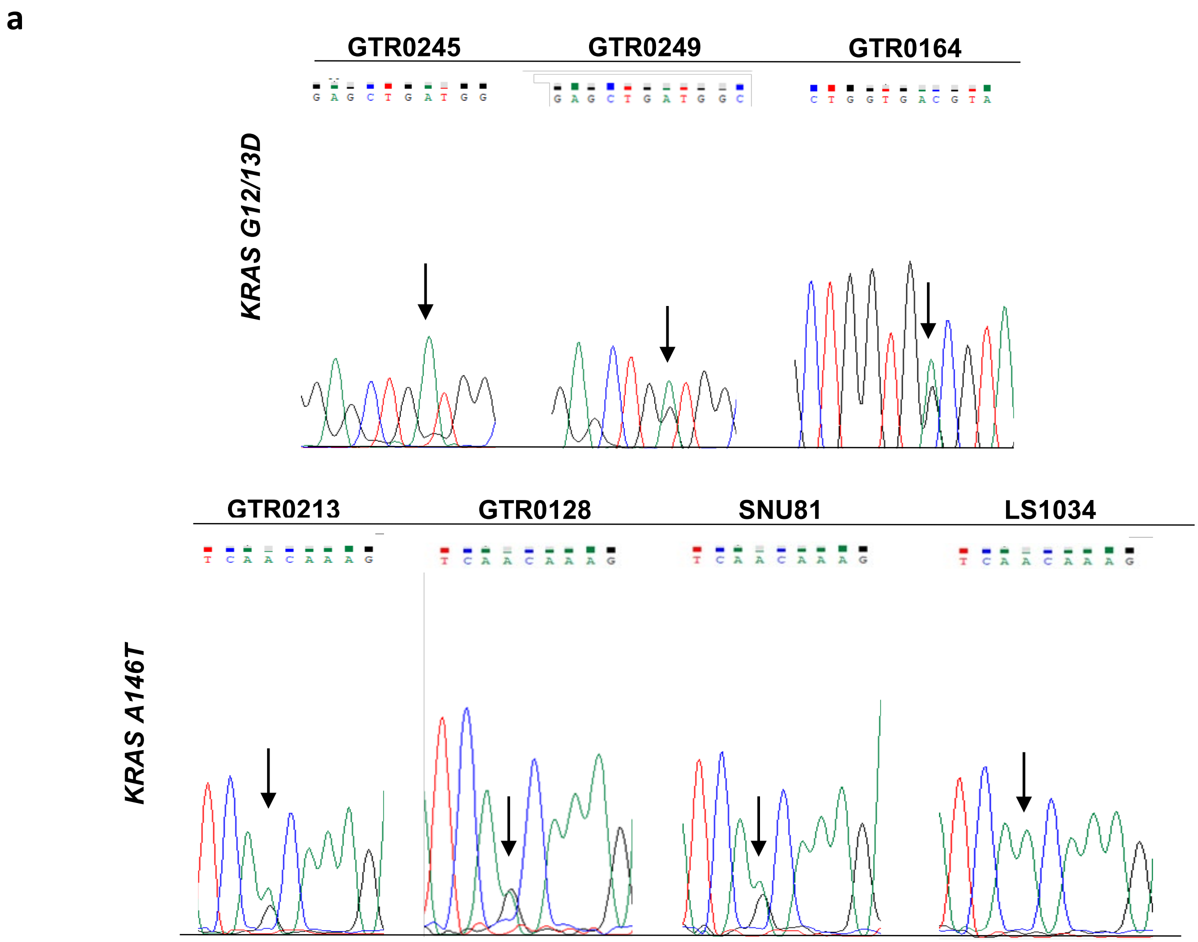

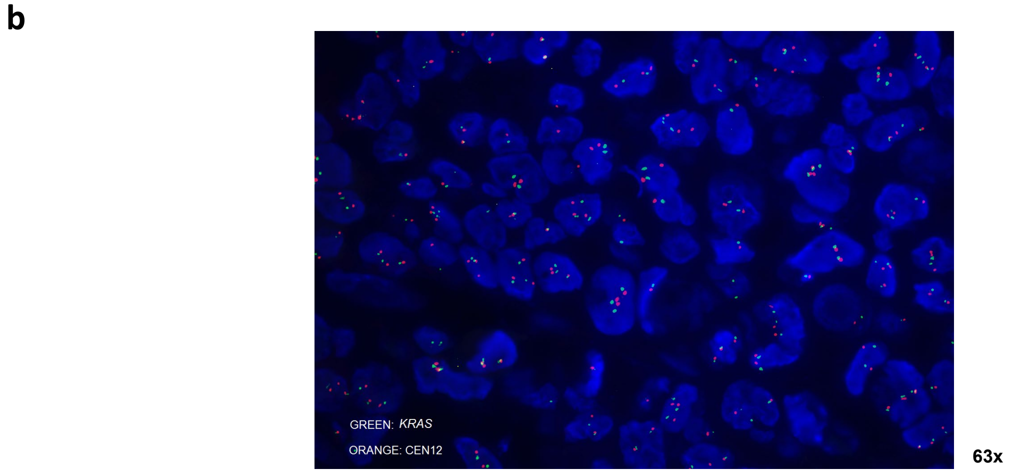

Supplementary Figure 1 *KRAS* gene mutations in Gastric Cancer PDX models used *in vitro* and *in vivo* experiments. (a) Electropherograms obtained by Sanger sequencing showing *KRAS* mutations in the different models. **(b)** *KRAS* fluorescence *in situ* hybridization for the GTR0245 PDX. *KRAS* (green) and CEN12 (red) signals (63x magnification).

Supplementary Figure 2

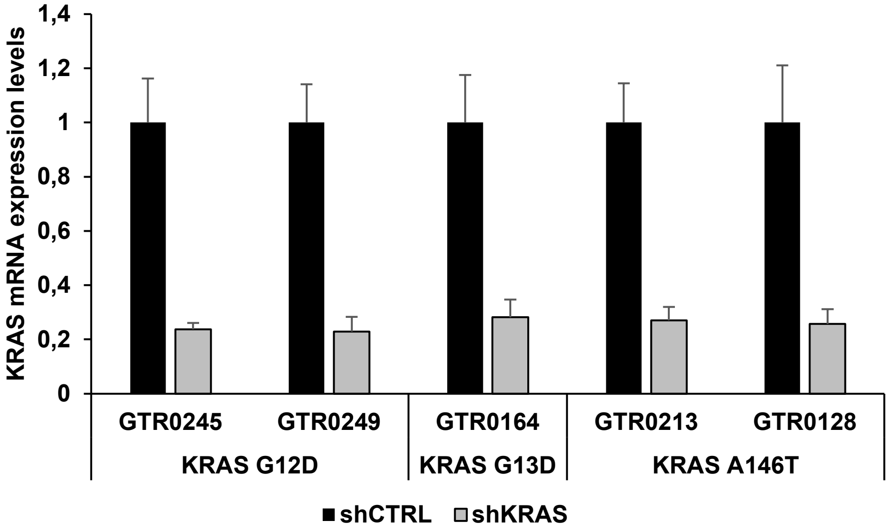

Supplementary Figure 2 Effectiveness of KRAS silencing. 48h after cell transduction with either control siRNA or KRAS siRNA, *KRAS* mRNA was quantified by qRT-PCR. The graph shows the percentage of expression in KRAS silenced cells vs controls.

Supplementary Figure 3

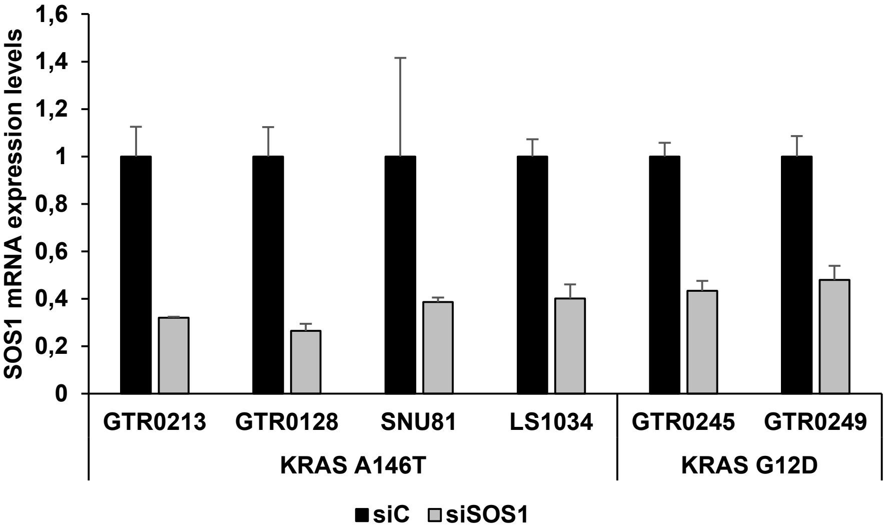

Supplementary Figure 3 Effectiveness of SOS1 silencing. The efficacy of *SOS1* silencing was demonstrated by measuring SOS1 mRNA levels by qRT-PCR 48h after transfection with either control siRNA or SOS1 siRNA.

Supplementary Figure 4

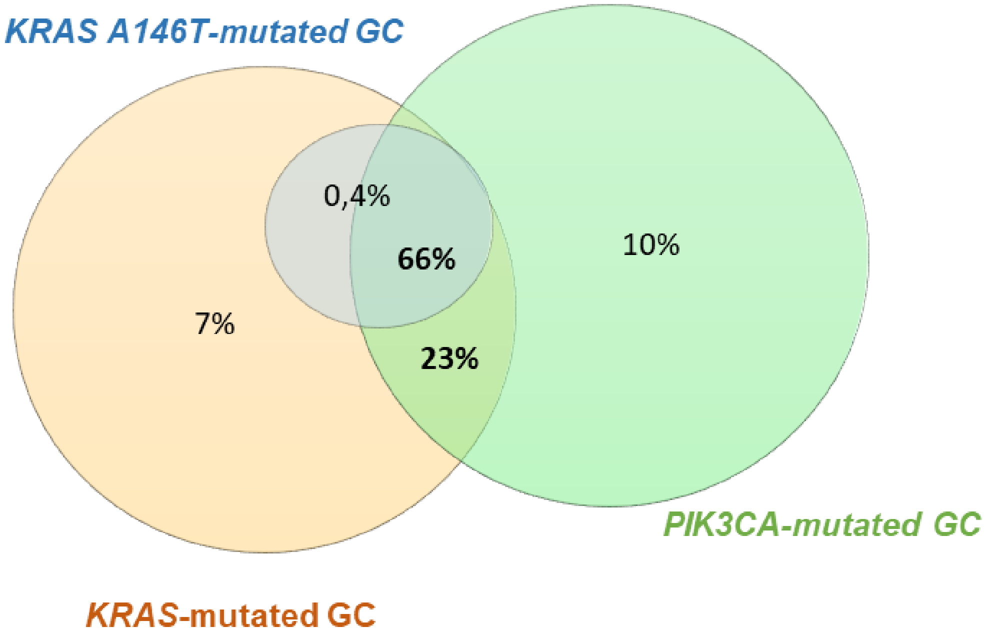

Supplementary Figure 4 Co-occurrence of KRAS and PI3KCA mutations in GC. Venn diagram showing the co-occurrence of canonical *KRAS*, *KRAS A146T* and *PI3KCA* mutations in a cohort of 777 gastric cancer patients. Five different gastric cancer databases were interrogated on cBioportal.
